# New trends in radiology education, time to adapt locally

**DOI:** 10.12669/pjms.36.COVID19-S4.2823

**Published:** 2020-05

**Authors:** Faheemullah Khan, Noman Khan, M. Arif Saeed

**Affiliations:** 1 Faheemullah Khan, MBBS. Department of Radiology, Aga Khan University Hospital, Karachi, Pakistan; 2 Noman Khan, MBBS. Department of Radiology, Aga Khan University Hospital, Karachi, Pakistan; 3 Muhammad Arif Saeed, FCPS. Department of Radiology, Northwest General Hospital & Research Center, Peshawar, Pakistan

The current COVID-19 pandemic has affected every aspect of life. The most affected is the healthcare system. Radiology forms an integral part of that system. Radiology practices are currently faced with multitude of problems. That includes how to provide continuous care to patients but also how to continue the medical education mission. Education is a core mission of residency programs. Program Directors are faced with a challenging job, deciding how can they best balance the competing demands of clinical work and the educational needs of their residents.

Globally the advanced healthcare centers are adapting in the wake of COVID-19 pandemic, sticking to the principle of social distancing while at the same time caring for their patients and continuing their radiology education mission. Diagnostic Radiology Residency programs of New York University Langone Health, University of Washington and Vanderbilt University Medical Center recently published what changes have they incorporated in their programs.^1^ Remote learning has become a norm at these advanced health care centers. Faculty and trainees adapted rapidly using screen-sharing software for teaching. Multiple radiology societies quickly organized and disseminated free learning material for residents on their official web forums.^2^ The radiology residency program at the Aga Khan University Hospital has pursued the same to achieve the program objective. Residents educational sessions are happening using the Microsoft Teams forum. Residents are tasked with images submission via email in the form of a quiz and they are then discussed in the online session.

Pakistan, where there is already shortage of quality radiologists, this mission must continue with the same zeal and spirit to educate the next generation of radiologists. This is the point where College of Physicians and Surgeons Pakistan (CPSP) and Radiological Society of Pakistan (RSP) have to play its role as parent bodies for the mission.

## Authors’ Contribution

**FK:** Conception, write up, critical review and responsible of study.

**NK and MAS:** Conception and critical review.

All the authors approved the final version.
